# Scale-Free Neural and Physiological Dynamics in Naturalistic Stimuli Processing

**DOI:** 10.1523/ENEURO.0191-16.2016

**Published:** 2016-10-24

**Authors:** Amy Lin, Brian Maniscalco, Biyu J. He

**Affiliations:** 1 National Institute of Neurological Disorders and Stroke, National Institutes of Health, Bethesda, MD 20892, USA; 2 Semel Institute for Neuroscience and Human Behavior, University of California-Los Angeles, Los Angeles, California 90095; 3 Neuroscience Institute, New York University Langone Medical Center, New York, NY 10016, USA; 4 Departments of Neurology, Neuroscience and Physiology, and Radiology, New York University Langone Medical Center, New York, NY 10016

**Keywords:** α oscillations, arrhythmic brain activity, heart rate variability, natural stimuli, scale-free dynamics, slow cortical potentials

## Abstract

Neural activity recorded at multiple spatiotemporal scales is dominated by arrhythmic fluctuations without a characteristic temporal periodicity. Such activity often exhibits a 1/*f*-type power spectrum, in which power falls off with increasing frequency following a power-law function: $P\left(f\right)\propto 1/f^{\beta }$, which is indicative of scale-free dynamics. Two extensively studied forms of scale-free neural dynamics in the human brain are slow cortical potentials (SCPs)—the low-frequency (<5 Hz) component of brain field potentials—and the amplitude fluctuations of α oscillations, both of which have been shown to carry important functional roles. In addition, scale-free dynamics characterize normal human physiology such as heartbeat dynamics. However, the exact relationships among these scale-free neural and physiological dynamics remain unclear. We recorded simultaneous magnetoencephalography and electrocardiography in healthy subjects in the resting state and while performing a discrimination task on scale-free dynamical auditory stimuli that followed different scale-free statistics. We observed that long-range temporal correlation (captured by the power-law exponent β) in SCPs positively correlated with that of heartbeat dynamics across time within an individual and negatively correlated with that of α-amplitude fluctuations across individuals. In addition, across individuals, long-range temporal correlation of both SCP and α-oscillation amplitude predicted subjects’ discrimination performance in the auditory task, albeit through antagonistic relationships. These findings reveal interrelations among different scale-free neural and physiological dynamics and initial evidence for the involvement of scale-free neural dynamics in the processing of natural stimuli, which often exhibit scale-free dynamics.

## Significance Statement

Many time-varying natural stimuli such as natural soundscapes, speech, and music exhibit scale-free dynamics characterized by a 1/*f*-type power spectrum. In parallel, scale-free neural dynamics are prominent across observational levels in the brain. Two well-established forms of scale-free neural activity are slow cortical potentials and amplitude fluctuations of α oscillations. However, it is unknown whether they are related. In addition, the interbeat interval fluctuation of the healthy human heart follows scale-free dynamics, but its relationship with scale-free neural dynamics is not fully characterized. We observed novel relationships between these different scale-free neural and physiological dynamics. Moreover, naturalistic stimuli exhibiting scale-free dynamics modulate scale-free neural dynamics, and baseline characteristics of scale-free neural dynamics predict an individual’s ability to process naturalistic stimuli.

## Introduction

**Figure 1.**
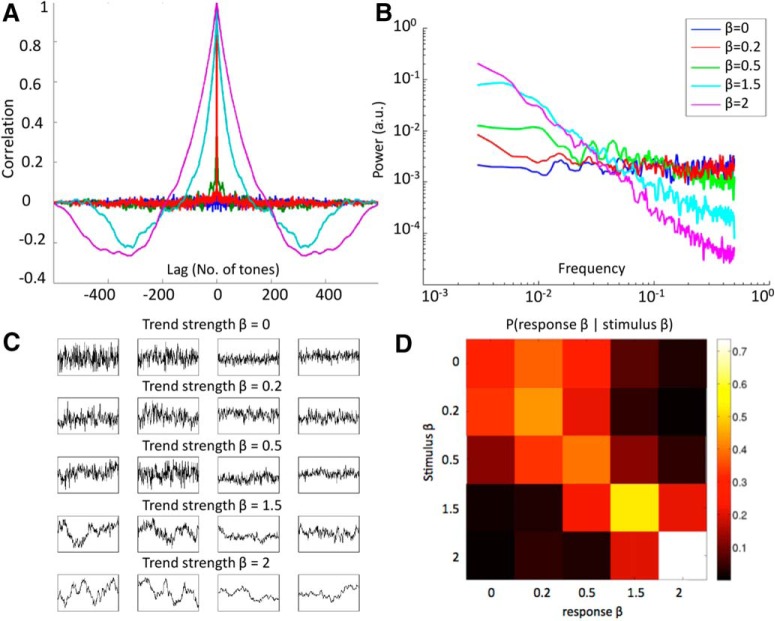
Stimuli characteristics and behavioral performance. ***A***, Lagged autocorrelation function for each class of stimulus sequences, averaged across the six examples at each β level. ***B***, Power spectra of stimulus sequences, averaged across the six examples at each β level. ***C***, Visual instruction presented to the subjects, showing example stimulus sequences at different β levels. Sequences of two different overall range were presented (two left columns, large fluctuation range; two right columns, small range), to demonstrate that trend strength (i.e., autocorrelation) is independent from overall range. ***D***, Group-average (*n* = 19) conditional probability of behavioral response given stimulus β, color-coded by the proportion of response β at each stimulus-β level. Each row sums to 1.

Many natural stimuli exhibit scale-free temporal or spatial patterns, such that no particular temporal or spatial periodicity predominates (Mandelbrot, 1999). In the spatial domain, it is well documented that natural images follow a $P\left(f\right)\propto 1/f^{\beta }$ spatial power spectrum, where *f* is the spatial frequency (Field, 1987). In the temporal domain, scale-free dynamics are characterized by a $P\left(f\right)\propto 1/f^{\beta }$ temporal power spectrum, where *f* is the temporal frequency and the power-law exponent β captures the strength of autocorrelation in the signal over time. In dynamics with a larger β, trends tend to persist over longer periods of time (Fig. 1*A–C*). Time-varying natural images (i.e., natural movies) typically follow a $P\left(f\right)\propto 1/f^{\beta }$ -type temporal power spectrum (Dong and Atick, 1995). Loudness and pitch fluctuations of natural soundscapes, such as urban and rural environmental noise (De Coensel et al., 2003), speech, and music (Voss and Clarke, 1975), also exhibit 1/*f*-type temporal power spectra.

In the brain, scale-free dynamics are prominent across multiple observational levels (He, 2014) and manifest in human behavioral output (Gilden, 2001). Two well-studied forms of scale-free neural dynamics are the slow cortical potentials (SCPs; He et al., 2010) and amplitude fluctuations of brain oscillations (Linkenkaer-Hansen et al., 2001). The SCPs are the low-frequency (<5 Hz) component of broadband field potentials that exhibit a 1/*f*-type power spectrum. Changes in arousal state and task performance alter the power-law exponent in the SCP range recorded by electrocorticography in humans (He et al., 2010). In addition, the SCP correlates with functional MRI (fMRI) signals in both spontaneous fluctuations and stimulus-driven responses (He et al., 2008; He and Raichle, 2009; Kahn et al., 2013; Pan et al., 2013), and the spontaneous fMRI signals exhibit prominent scale-free dynamics (He, 2011).

A parallel line of research has established that amplitude fluctuations of brain α oscillations also follow scale-free dynamics (Linkenkaer-Hansen et al., 2001). Its power-law exponent has been shown to vary with task performance (Linkenkaer-Hansen et al., 2004), have a genetic contribution (Linkenkaer-Hansen et al., 2007), increase during development (Smit et al., 2011), and differ between patients with Alzheimer’s disease and controls (Montez et al., 2009). Moreover, the power-law exponent of α-oscillation amplitude predicts that of behavioral fluctuations across individuals (Palva et al., 2013; Smit et al., 2013), indicating a link between neural and behavioral long-range temporal correlation.

Although existing data suggest that SCP phase modulates α-oscillation amplitude (He, 2014), whether scale-free dynamics in the SCP and α-amplitude fluctuations are related remains unknown. In addition, a rich literature establishes that the healthy human heart is characterized by scale-free dynamics in its interbeat interval, and failure thereof accompanies heart disease or aging (Goldberger et al., 2002). Currently, the literature remains mixed about whether and how scale-free dynamics in the SCP and α amplitude interact with heartbeat dynamics (Palva et al., 2013; Zhigalov et al., 2015). This question is especially intriguing in light of recent data showing that afferent signals from the heart interact with not only the limbic system but also perceptual and cognitive systems in the brain (Park et al., 2014).

We recorded simultaneous magnetoencephalography (MEG) and electrocardiography (ECG) in human subjects in the resting state and while listening to auditory tone sequences whose pitch fluctuations constituted scale-free dynamics with varying degrees of autocorrelation. These auditory stimuli captured second-order statistics in natural stimuli, since their temporal power spectra followed a power-law distribution. Subjects discriminated tone sequences with different degrees of autocorrelation, as captured by the power-law exponent β. We investigated the relationships among scale-free neural and heartbeat dynamics across sensors and individuals and over time within an individual. We further examined whether the degree of autocorrelation in scale-free auditory stimuli modulated scale-free neural and heartbeat dynamics, and whether scale-free neural dynamics predicted an individual’s ability to discriminate stimuli with different degrees of autocorrelation. We hypothesized, first, that scale-free dynamics in the SCP, α-amplitude fluctuations, and heartbeat dynamics are interrelated, and second, that scale-free neural dynamics are involved in the processing of dynamic, scale-free stimuli.

## Materials and Methods

### Subjects

The experiment was approved by the Institutional Review Board of the National Institute of Neurological Disorders and Stroke. All subjects were right-handed and neurologically healthy with normal hearing. Nineteen subjects between 19 and 30 years old (mean age 24.7; 12 females) participated in a ∼3-h long MEG session with simultaneous ECG recording. Two subjects did not have ECG data because one subject was tested before the ECG recording was implemented, and the other subject’s ECG data was too noisy to reliably extract R peaks. All subjects provided written informed consent.

### Stimulus creation

We created auditory tone sequences whose pitch fluctuations had five levels of autocorrelation strength, spanning from fractional Gaussian noise (fGn) to fractional Brownian motion (fBm). We used a circulant embedding algorithm (Helgason et al., 2011) to create fGn time series with Hurst exponents of 0.5, 0.6, and 0.75 (corresponding to power-law exponent β = 0, 0.2, and 0.5, respectively, where β = 2*H* – 1), as well as fBm time series with Hurst exponents of 0.25 and 0.5 (corresponding to power-law exponent β = 1.5 and 2, where β = 2*H* + 1). We created six unique 600-element long series for each level of β,

$${\underline{x}}_{\beta }=\left[x_{1},x_{2},\ldots ,x_{600}\right],\beta \in \left\{0,0.2,0.5,1.5,2\right\},1\leq i\leq 6,$$

where each element *x_j_* of **x**_β,_*_i_* is taken to represent the pitch of the *j*th tone in the sequence. We verified that each synthesized **x**_β,_*_i_* indeed had the desired β by computing the autocorrelation function and performing power spectral analysis for each sequence (Fig. 1*A* and 1*B*, respectively). Each auditory sequence *i* was unique to ensure that subjects would respond to statistical properties of the sequence rather than memorizing particular features.

After verifying the autocorrelation properties of the sequences, we translated and scaled each **x**_β,_*_i_* so that its elements ranged from log(220) to log(880) and discretized the series such that each element took on one of 25 values evenly spaced on the log scale. Let us refer to the scaled, translated, and discretized series as **p**_β,_*_i_* . Each **p**_β,_*_i_* thus represents a time series of tone pitches, where pitch varies in semitone steps between 220 and 880 Hz and exhibits autocorrelation prescribed by β. This range of pitch values was chosen to span the isoloudness region of human hearing (i.e., with identical amplitude, subjective loudness varies minimally with changing pitch in this range).

To produce an auditory stimulus for each tone sequence, we first computed the time series of tone frequencies as ${\underline{f}}_{\beta ,i}=\exp\left({\underline{p}}_{\beta ,i}\right)$, in Hz. For each $f_{j}\in {\underline{f}}_{\beta ,i}$, we constructed a sinusoidal sound wave of duration 300 ms at a 44,100-Hz sampling frequency (thus yielding 13,230 samples for each tone) according to

$${\underline{y}}_{j,s}=A\cos\left[2\pi f_{j}\left(s/SR\right)+\varphi_{j}\right],1\leq s\leq 13,230,$$

where *s* denotes sample number, *f_j_* denotes tone frequency, *SR* denotes the sampling frequency of 44,100 Hz, and *A* = 1. The amplitude of the tones was kept identical throughout the sequence, i.e., the tones were not amplitude-modulated. The 300-ms duration was chosen for ease of listening (Patel and Balaban, 2000). Because each tone was 300 ms and each sequence contained 600 tones, each sequence had a total duration of 180 s.

Cosine waves ${\underline{y}}_{j,s}$ for each tone *j* were concatenated, such that there was no silence period between consecutive tones. For the first tone *j* = 1, the phase φ was set to 0. For subsequent tones *j* > 1, the absolute value of φ was set to the arccosine of the final sample of tone *j* – 1. The sign of φ for tone *j* was set such that the first-order time derivative was continuous across the transition from the end of tone *j* – 1 to the start of tone *j*. This ensured that there was a smooth and continuous transition between cosine waves in the junctions where tone frequency changed.

Auditory sequences were presented using the PsychPortAudio function of the Psychophysics Toolbox (Brainard, 1997) in Matlab (Mathworks, Natick, MA). The audio was delivered through specialized ear tubes that were custom fitted to work within the MEG scanner. We used Etymotic ER-3 Insert Headphones, in which the frequency response is flat to 5 kHz. The plastic tubing from the transducer to the earpiece had a speed-of-sound delay of around 10 ms, which was corrected in MEG data analyses.

### Experimental design

After presentation of each auditory tone sequence (3 min long), subjects were asked to judge its “trend strength” on a scale of 0 to 4. Higher “trend strength” was explained to the subjects as the tendency for a trend to persist over longer period of time, which captured the strength of autocorrelation in the time series. Ascending entries on the “trend strength” scale corresponded to ascending levels of stimulus β. Visual performance feedback was presented after every trial to assist subjects in learning how to use the scale to accurately characterize stimulus β. The feedback indicated what trend strength rating had been entered by the subject, what the true trend strength of the sequence was, and whether the subject’s trend strength rating was correct, close to correct (off by one level of trend strength), or incorrect (off by two or more levels of trend strength). To ensure adequate task performance, all subjects were trained during an initial behavioral session that took place at least a few days previously in which shortened versions of stimuli sequences were used. Subjects who performed adequately (behavioral ρ > 0.2, see below) were invited back for the MEG/ECG experiment (82.4% of all subjects tested). At the start of the initial behavioral session as well as the main MEG/ECG experiment, subjects were visually presented with shortened examples of each level of trend strength, as exemplified in Fig. 1*C*, which shows that trend strength was independent of overall range. Subjects also completed one practice block at the start of the main experiment.

Stimulus sequences were initially created with the intention of having β values of 0, 0.5, 1.01, 1.5, and 2, and feedback presented to the subject was consistent with this scheme. However, because of an aliasing artifact, stimuli with an intended β = 1.01 had an empirical β closer to 0.2 (Fig. 1*A*,*B*), and so we treat these stimuli as having β = 0.2 in all analyses. Because of this complication, the implicit mapping between trend strength rating and stimulus β communicated to subjects via visual feedback was erroneous for the β = 0.2 case. Nonetheless, subjects demonstrated an ability to accurately detect that pitch autocorrelation was weaker for stimulus β = 0.2 than for β = 0.5 (Fig. 1*D*). This suggests that subjects were able to accurately classify stimulus β in spite of the feedback error and further justifies our treatment of these stimuli as β = 0.2 rather than β = 1.01. Additionally, we verified that no analysis presented herein yielded different statistical inference if using the originally intended value of stimulus β = 1.01 rather than the empirically derived value of β = 0.2.

We also included a rest condition (3-min long trials) in which no auditory stimulus was presented. Throughout the experiment—during both auditory task and resting state—subjects were asked to keep their gaze fixed on a cross presented at the center of the screen to minimize eye movement. Each subject completed 36 trials in total, which included six trials per condition (five stimulus β levels plus rest condition). The stimulus sequences differed across trials within the same β level. The trials were grouped in blocks of three, resulting in 12 blocks in total. Stimulus β was randomized across blocks, whereas rest trials were evenly dispersed throughout the experiment (always presented as the second trial of even numbered blocks). The head position of the subject was measured with respect to the MEG sensor array using coils placed on the left and right preauricular points and the nasion. Before each block subsequent to the first block, the subject self-corrected the head position to the same position recorded at the start of the first block using a custom visual-feedback program to minimize head displacement across the experiment. Video monitoring of the subject during the experiment ensured that subjects stayed alert and did not close their eyes for extended periods of time. After excluding trials that had failure of behavioral response or drowsiness as shown by eye closure, 14 subjects had all 36 trials, two subjects had 35 trials, two subjects had 33 trials, and one subject had 30 trials.

### Data acquisition and preprocessing

Experiments were conducted in a whole-head 275-channel CTF MEG scanner (VSM MedTech, Coquitlam, BC, Canada). MEG data were collected with a sampling rate of 600 Hz and an anti-aliasing filter at <150 Hz. Analyses were performed on 271 sensors after excluding four malfunctioning sensors. The Fieldtrip package (Oostenveld et al., 2011) implemented in Matlab was used for data preprocessing, and analyses were conducted using Fieldtrip and custom-written code. We used independent component analysis to remove artifacts related to eye blinks, eye movements, heartbeat, breathing, and slow movement drift. Empty-room recording was collected in a prior experiment to verify that instrument noise was orders of magnitude lower than the signal we analyzed.

### Measuring scale-free parameters

**Figure 2.**
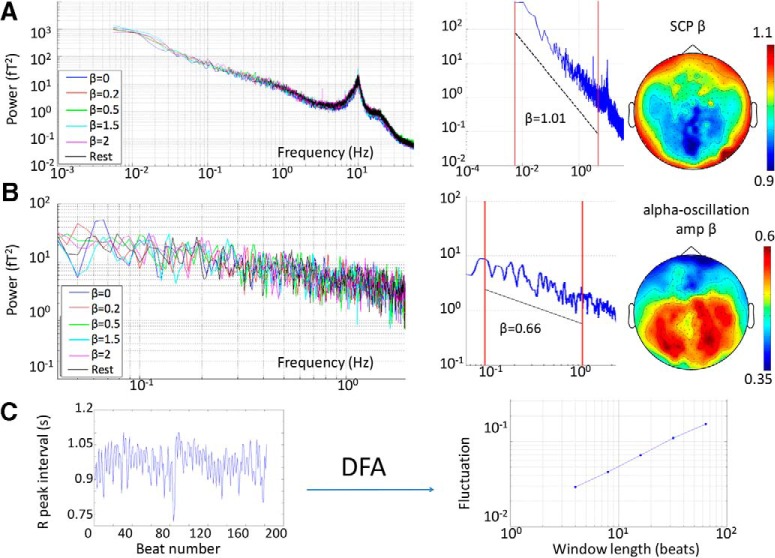
Characterization of neural and physiological dynamics. ***A***, Left, MEG signal power spectra from an example subject in each task condition (averaged across all sensors and trials within a condition). Middle, example MEG signal power spectrum from a single trial in one subject. The red lines indicate the frequency range for extracting SCP power-law exponent β: 0.005–5 Hz. Right, grand-average topographic map of SCP β across the scalp. ***B***, As in ***A***, but for α-oscillation amplitude. α Oscillation was filtered in the 6.7- to 13.3-Hz range, and its amplitude time series was extracted using the Hilbert transform. α-Amplitude β was extracted in the 0.1- to 1-Hz range (red lines in the middle panel). ***C***, For analysis on heartbeat dynamics, the interbeat interval was calculated as the difference in time (s) between adjacent R-peaks in the ECG recording (left panel). The interbeat interval time series was subjected to DFA to extract the DFA exponent α, which describes the power-law relationship between fluctuation magnitude and the length of observation in scale-free dynamics (right panel).

Various scale-invariance measures are mathematically related and thus can be reasonably compared (Eke et al., 2002). Power-law exponents of SCP and α-oscillation amplitude fluctuations were estimated using the common power spectral analysis. To estimate the power-law exponent β of the SCP, a fast Fourier transform was applied to the MEG signal from each sensor in each trial (3 min long) to compute its power spectrum. The power spectrum was plotted in double-logarithmic scale (Fig. 2*A*). Because a power spectrum following $P\left(f\right)\propto 1/f^{\beta }$ can be rewritten as log[P(f)]−βlog(f), the negative slope in the log–log plot provides a convenient measure of the power-law exponent β. In line with previous studies (He et al., 2008, 2010), the SCP β was estimated in the range of 0.005–5 Hz (power spectra were calculated on each trial lasting 180 s; thus the lowest frequency visible was 0.0056 Hz).

To estimate the power-law exponent β of α-oscillation amplitude fluctuations, we first extracted α oscillations from continuous MEG signal in each trial using a third-order Butterworth filter between 6.7 and 13.3 Hz and computed its instantaneous amplitude envelope by applying the Hilbert transform. A fast Fourier transform was then applied to the α-oscillation amplitude fluctuation to create a power spectrum for each sensor in each trial. The α-oscillation amplitude β was estimated from the log-log plot of power spectrum using the 0.1- to 1-Hz range (Fig. 2*B*).

Following previously established methodology, we applied detrended fluctuation analysis (DFA) on heartbeat dynamics (Goldberger et al., 2002; Hardstone et al., 2012). This computation was applied to the time variation in interbeat interval, measured as the interval in seconds between adjacent R peaks (Fig. 2*C*, left). DFA analysis was conducted as follows. First, the interbeat interval time series from each trial was integrated, and the mean was subtracted. We then estimated the local trend in nonoverlapping windows of equal length using a least-squares fit and determined the fluctuation as variance on the local trend for a given window. Five different window lengths were used: 4, 8, 16, 32, and 64. The log-log plot of mean fluctuation (*F*) against window length (*l*) constitutes the DFA plot (Fig. 2*C*, right), and its slope estimates the DFA exponent α, following the relation: $F\left(l\right)\propto l^{\alpha }$, or log[F(l)]∝αlog(l). Theoretically, the power-law exponent β is related to the DFA exponent α, following β=2α−1 (Eke et al., 2002).

### Interrelations between scale-free neural and heartbeat dynamics

**Figure 3.**
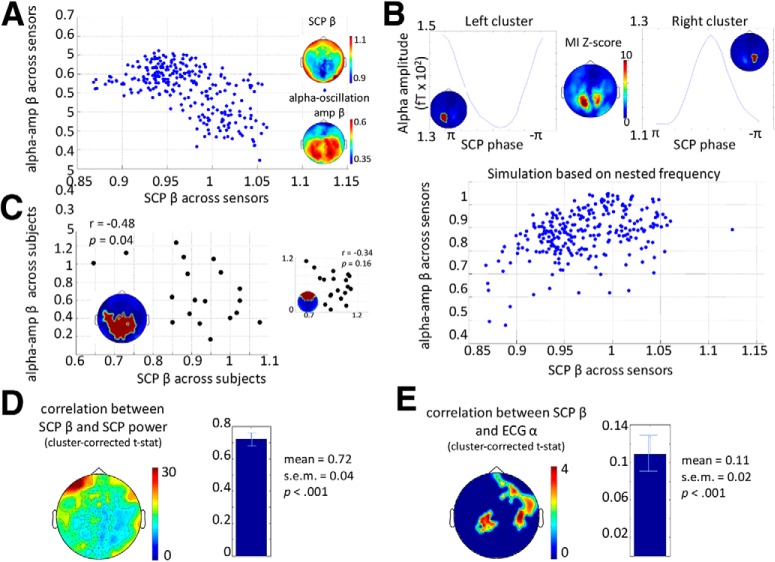
Interrelations between scale-free neural and physiological dynamics. ***A***, Scatterplot of SCP β against α-amplitude β across all MEG sensors (averaged across subjects and stimulus conditions). The inset shows the grand-average topographical plots of SCP β and α-amplitude β, reproduced from Fig. 2. ***B***, Top, nested-frequency analysis between SCP phase and α amplitude. Group-average MI *z*-score topography plot is shown (middle), along with the phase-amplitude histogram for the two dominant clusters (see insets). Bottom, scatterplot between empirically measured SCP β and simulated α-oscillation amplitude β across all sensors. ***C***, Left, scatterplot across subjects between SCP β and α-amplitude β in a posterior sensor cluster (see inset). Right, scatterplot across subjects between SCP β and α-amplitude β in an anterior sensor cluster (see inset). ***D***, Correlation between SCP β and SCP power across trials within an individual. Pearson correlation values were Fisher’s *z* transformed and subjected to a group-level one-sample *t*-test. *t* Values are plotted in the topography plot, with a single cluster encompassing all sensors surviving cluster-based correction. The group-level mean and SEM of Fisher *z* values (averaged across all sensors) are shown in the bar graph to the right (*p* < 0.001, one-sample *t*-test across subjects). ***E***, Similar to ***D***, but for within-subject, across-trial correlation between SCP β and ECG α. Two clusters of sensors survived cluster-based correction. The group-level mean and SEM of Fisher *z* values (averaged across all significant sensors) are shown in the bar graph to the right (*p* < 0.001, one-sample *t*-test across subjects).

To qualitatively assess the relationship between SCP β and α-oscillation amplitude β across sensors, we first generated grand-average scalp topography for SCP β and α-amplitude β across subjects and trials (Fig. 2*A*,*B*, right). No formal statistical test was performed for the relationship between SCP β and α-amplitude β across all sensors due to the difficulty in accurately accounting for the degrees of freedom associated with the sensors. However, a scatterplot of SCP β and α-amplitude β across sensors is included for descriptive purposes (Fig. 3*A*).

To investigate the relationship between SCP β and α-amplitude β across subjects, we first defined two clusters of sensors based on the topographic map of SCP β (Fig. 3*C*) and then averaged SCP β and α-amplitude β, respectively, across sensors within each cluster. Pearson’s correlation was calculated between SCP β and α-amplitude β across subjects for each of the two clusters.

To assess the relationship between any two of our three scale-free parameters (SCP β, α-amplitude β, or ECG α) over time, we calculated Pearson’s correlation between them across all 36 trials within each subject. Pearson’s *r* values were transformed into Fisher’s *z*-values, which were subjected to a one-sample *t*-test across subjects. Statistical significance was assessed by a cluster-based nonparametric permutation test (Nichols and Holmes, 2002; Maris and Oostenveld, 2007). To this end, we shuffled one variable across trials for 1,000 iterations. For each iteration, Pearson’s correlation between the two variables was recomputed, and the *r*-value was transformed into Fisher’s *z*-value and submitted to a one-sample *t*-test across subjects as with the original data. Clusters were defined for both the original and shuffled data as contiguous groups of sensors with *p*-values less than 0.05 and *t*-values of the same sign. Summing the *t*-values created a summary measure of each cluster. To build the null distribution, the absolute value of the summed *t*-statistic of the largest magnitude was extracted from each iteration. Finally, the absolute magnitude of the summed *t*-statistic in each cluster from the original data was compared to the null distribution. A cluster survived cluster-based correction if 2.5% or fewer of observations in the null distribution surpassed the absolute value of the cluster’s summed *t*-statistic (corresponding to *p* < 0.05 in a two-tailed test). Correlation between MEG signal power and β was tested similarly.

### SCP: α-oscillation nested-frequency analysis and simulation

Given previous EEG and electrocorticography findings showing a nested-frequency relationship between SCP phase and α-oscillation amplitude (Vanhatalo et al., 2004; Monto et al., 2008; He et al., 2010; He, 2014), a natural question is whether this nested-frequency relationship produces any correlation between SCP β and α-amplitude β. To address this question, we performed simulations to reveal what kind of relationship between SCP β and α-amplitude β would be expected if it were driven entirely by the nested-frequency relationship between them.

First, we quantified the nested-frequency relationship between SCP phase and α-oscillation amplitude using the well-established modulation index (MI; Tort et al., 2008; He et al., 2010). We extracted the SCP phase time series by using a third-order Butterworth filter between 0.005 and 1 Hz (using a 0.005- to 5-Hz filter yielded nearly identical results) and then applying the Hilbert transform. The α-oscillation amplitude time series was derived as described above. SCP α nested-frequency plots (for each subject, sensor, and condition) were generated by binning the SCP phase time series into 20 evenly spaced phase bins and averaging the α-oscillation amplitude within each phase bin. The MI was computed based on this nested-frequency plot, which uses an inverted entropy measure to quantify its deviation from a uniform distribution. For statistical testing, the MI value was converted into an MI *z*-score by comparison with a null distribution generated by shuffling the phase time series using five equal-length segments, following a previously described method (He et al., 2010). A preliminary analysis suggested that stimulus condition did not modulate MI *z*-score, and thus different conditions were combined in the subsequent simulation.

We next used the empirically measured SCP phase series in conjunction with the nested-frequency relationship between SCP phase and α-oscillation amplitude at each sensor to construct simulated α-oscillation amplitude time series. To this end, the SCP-α nested-frequency plot as described above was averaged across subjects and conditions for each sensor. This distribution was smoothed using a five-bin-wide moving average. For each sample of the SCP phase time series, we used spline interpolation of the nested-frequency distribution at that sensor to determine what α-oscillation amplitude would be predicted by the SCP phase. Finally, we computed the β of the empirical SCP time series and the simulated α-oscillation amplitude series, averaged these β values over trials and subjects, and examined their Pearson correlation across sensors. This result reveals the contribution of the nested-frequency relationship between SCP and α oscillations to the correlation between SCP β and α-oscillation amplitude β.

### Stimulus modulation of scale-free neural and heartbeat dynamics

We probed whether the strength of autocorrelation in the auditory stimulus (as captured by its power-law exponent β) modulated scale-free neural and heartbeat dynamics. For each sensor in each subject, stimulus β was correlated with SCP β, α-oscillation amplitude β, or ECG α across the 30 task trials using Spearman’s rank correlation. Spearman’s rho values were transformed into Fisher’s *z*-values (Fieller et al., 1957) and submitted to a one-sample *t*-test across subjects at each sensor. Statistical significance was established using a cluster-based nonparametric permutation test as described earlier.

### Behavioral performance assessment and correlation with scale-free neural dynamics

To evaluate subjects’ behavioral performance in the stimulus β discrimination task, we first visualized the conditional probability of behavioral response β (i.e. the β corresponding to the subject’s trend strength rating) at a given stimulus β (Fig. 1*D*). The behavioral performance of each subject was assessed by Spearman’s rank correlation between stimulus β and response β across all task trials. The Spearman’s ρ, or behavioral ρ, captures a subject’s behavioral performance for the entire experiment while allowing for some leniency in which subjects could be close to the right answer but not exact. To test whether scale-free neural dynamics predicted behavioral performance on a subject-by-subject basis, we computed Pearson’s correlation between behavioral ρ and either SCP β or α-oscillation amplitude β at each sensor across subjects.

## Results

### Behavioral performance

The across-trial Spearman’s correlation between stimulus β and response β (“behavioral ρ”) was significant for every subject (*n* = 19, *p* ranged from *p* < 0.0001 to 0.048 [Table 1, line a]), indicating that all subjects could perform the stimulus β discrimination task significantly above chance level. Behavioral ρ ranged from 0.36 to 0.88, with an average value of 0.66. Fig. 1*D* shows the group-average conditional probability map of behavioral response given stimulus β. The accuracy of subjects’ behavioral responses is reflected by the concentration of the probability distribution along the diagonal, where response β is equal to stimulus β. Note that incorrect behavioral responses tended to be close to the correct response (i.e. off-diagonal elements that are closer to the diagonal have higher probability than those that are farther away). The Spearman’s correlation coefficient thus provides a more informative and natural measure for quantifying overall behavioral performance than proportion correct. In particular, Spearman’s correlation, but not the proportion of correct responses, takes into account the magnitude of response error. Interestingly, subjects were better at discriminating between stimulus β in the fBm (β = 1.5 or 2) than the fGn range (β = 0, 0.2 or 0.5; Fig. 1*D*). We note that stimuli in the fBm range tended to sound more melodic than those in the fGn range.

**Table 1.**
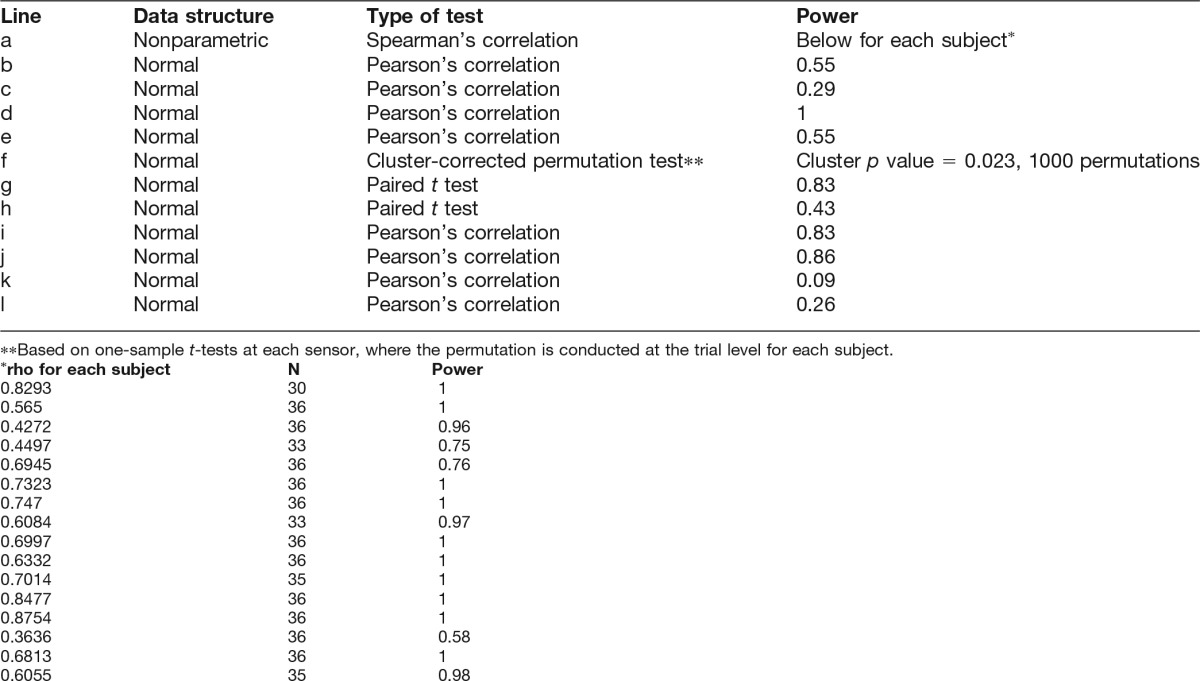
Statistical analysis.

### Scale-free dynamics in neural activity and heart rate variability

Consistent with earlier reports (Dehghani et al., 2010; He, 2014), the power spectrum of MEG signals followed power-law scaling, with peaks at discrete frequencies corresponding to various brain oscillations (see Fig. 2*A*, left, for example power spectra from a single subject). We extracted power-law exponent β for SCP from the 0.005- to 5-Hz frequency range (Fig. 2*A*, middle). The grand average (across stimulus conditions and subjects) topographical distribution of SCP β across MEG sensors is shown in Fig. 2*A* (right). SCP β ranged from 0.87 to 1.12 across sensors (mean 0.97), and exhibited an anterior-posterior gradient with frontal sensors displaying higher β, and hence longer temporal autocorrelation in the SCP. This finding is consistent with previous MEG (Dehghani et al., 2010) and fMRI (He, 2011) observations.

We extracted instantaneous amplitude fluctuations of α oscillations (filtered in the 6.7- to 13.3-Hz range), and computed its power spectrum (see Fig. 2*B*, left, for result from an example subject). In line with previous reports (Linkenkaer-Hansen et al., 2001), α-oscillation amplitude fluctuations exhibit power-law scaling in the power spectrum. We extracted the power-law exponent β of α amplitude in the range of 0.1–1 Hz (Fig. 2*B*, middle). α-Amplitude β ranged from 0.32 to 0.61 across sensors (mean 0.51) and displayed an anterior-posterior gradient opposite to that of SCP, such that posterior sensors had higher β, and accordingly, stronger autocorrelation (Fig. 2*B*, right).

Finally, following established procedures for analyzing heartbeat dynamics (Goldberger et al., 2002), we defined R-peaks from ECG recordings and constructed an interbeat interval time series, which was subjected to DFA analysis to extract the DFA exponent α (Fig. 2*C*). Theoretically, the DFA exponent α is directly related to the power-law exponent β (see Materials and Methods), both of which capture the strength of autocorrelation in a time series, one using a time-domain approach (DFA exponent α), the other a frequency-domain approach (power-law exponent β). Across 17 subjects with simultaneous ECG-MEG recordings, ECG α ranged from 0.57 to 1.2, with a mean of 0.82 across subjects, consistent with previous reports (Goldberger et al., 2002).

### Anticorrelation between SCP and α-oscillation amplitude power-law exponents

We next explored the relationship between scale-free dynamics in SCP and α-oscillation amplitude fluctuations across the scalp and subjects. Because stimulus condition minimally modulated power-law exponent of SCP or α-oscillation amplitude (see below), for this analysis, we pooled data across all conditions (including five stimulus β levels and resting condition).

The scalp topography of SCP β and α-oscillation amplitude β (Fig. 2*A*,*B*, right) display opposite anterior-posterior gradients, indicating that as the strength of autocorrelation in SCP increases, the strength of α-oscillation amplitude fluctuations tends to decrease. To qualitatively assess this relationship, we plotted the two measures, each averaged over 19 subjects, against each other across all sensors (Fig. 3*A*). This revealed a negative relationship between SCP β and α-oscillation amplitude β across MEG sensors.

We then assessed whether there might also be an anticorrelation between SCP β and α-amplitude β across subjects. To this end, we first defined two clusters of sensors based on the scalp topography of SCP β, distributed over frontal and posterior regions, with relatively high and low β, respectively. We then assessed across-subject correlation between SCP β and α-oscillation amplitude β for each cluster of sensors. In the posterior cluster, we observed a significant negative correlation between SCP β and α-amplitude β across subjects (Fig. 3*C*; *r* = –0.48, *p* = 0.037 [Table 1, line b], *n* = 19). In the frontal cluster, there was a negative trend that was not significant (Fig. 3*C*, inset; *r* = –0.34, *p* = 0.16 [Table 1, line c]). Could the significant anticorrelation between SCP β and α-oscillation amplitude β in the posterior cluster across subjects be driven by a relation between their respective power? Two pieces of evidence suggest that this is not the case. First, SCP power and α-oscillation power were found to correlate positively across subjects (*r* = 0.91, *p* = 8.5 × 10^–8^ [Table 1, line d]; but note that this finding in itself could be due to measurement variation across subjects). Second, a partial correlation analysis revealed that after controlling for the effects of SCP and α-oscillation power, the anticorrelation between SCP β and α-amplitude β across subjects in the posterior sensor cluster was unchanged (*r* = –0.48, *p* = 0.05 [Table 1, line e]).

The above results reveal an intriguing negative relationship between SCP β and α-amplitude β across the scalp and subjects, such that stronger autocorrelation in the SCP is accompanied by weaker autocorrelation in the amplitude fluctuations of α oscillations. In light of previous observations of a nested-frequency relationship between SCP phase and α-oscillation amplitude (Vanhatalo et al., 2004; He, 2014), these findings raise a natural question: is the anticorrelation between SCP β and α-amplitude β driven by the nested-frequency relationship between them? To test this hypothesis, we quantified the nested-frequency pattern between SCP phase and α-oscillation amplitude in each MEG sensor (Fig. 3*B*, top) and simulated α-oscillation amplitude time series for each sensor in each subject, based on the sensor-specific nested-frequency pattern and the empirically measured SCP phase time series. We then computed the power-law exponent β of the simulated α-oscillation amplitude time series and plotted it against the measured SCP β across all sensors (Fig. 3*B*, bottom). This simulation reveals a positive relationship between SCP β and α-amplitude β, suggesting that the negative relationship observed in the empirical data cannot be explained by the nested-frequency relationship between SCP and α oscillations.

Finally, we investigated whether the amount of power in the SCP or α range was related to their respective β across time within an individual (see Methods). We found a robust positive correlation between SCP power and β: after cluster-based correction for multiple comparisons, all MEG sensors across the entire scalp demonstrated a significant positive correlation (Fig. 3*D*). By contrast, α-oscillation power had no significant correlation with its amplitude β after cluster-based correction. This result is consistent with previous findings showing that SCP β changes by modulating the power in the lowest frequency ranges, thereby causing a positive correlation between its power and β (He et al., 2010).

### Relationship between scale-free neural and physiological dynamics

We further investigated whether the strength of autocorrelation in scale-free neural and heartbeat dynamics comodulated across time within an individual. To this end, we computed correlations between SCP β and the DFA exponent α of heartbeat dynamics measured by ECG. This analysis revealed two significant clusters, one over the left central cortex, and the other over the right central cortex extending into frontal areas (Fig. 3*E*). No significant correlation was found between α-amplitude β and ECG α after correction for multiple comparisons.

### Stimulus condition modulates scale-free dynamics in α-oscillation amplitude

Does the strength of autocorrelation in the stimulus sequence (“stimulus β”) modulate the strength of autocorrelation within scale-free neural or physiological dynamics? To answer this question, we computed Spearman’s rank correlation between stimulus β and the autocorrelation parameter from neural or heartbeat dynamics (respectively, SCP β, α-oscillation amplitude β, and ECG α) across all trials during the auditory task (30 trials in total) for each subject.

**Figure 4.**
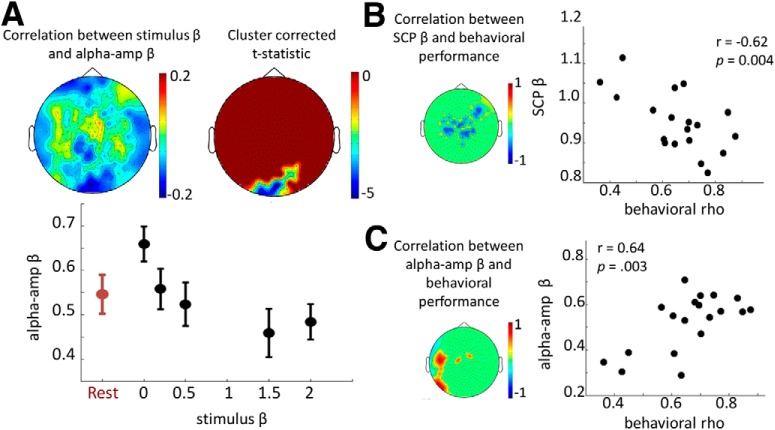
Stimulus modulation of scale-free neural dynamics and prediction of behavioral performance. ***A***, Spearman rank correlation was calculated between stimulus β and α-amplitude β across the 30 task trials for each subject, and the group average is plotted for all sensors (top left panel). A posterior sensor cluster survived cluster-based correction at *p* < 0.05 (top right panel). For this significant sensor cluster, α-amplitude β averaged across sensors was plotted for each stimulus β level and rest condition (bottom panel), which shows the mean and SEM across subjects. ***B***, Left, Pearson’s correlation value between behavioral performance (measured as behavioral ρ) and SCP β (averaged across all conditions) across subjects, thresholded at a *p* < 0.05 level. Nonsignificant sensors are shown as a uniform green background. Right, SCP β averaged across significant sensors is plotted against behavioral ρ for all subjects. ***C***, Same as ***B***, but for the correlation between α-amplitude β and behavioral performance.

We found that as stimulus β increased, α-amplitude β progressively decreased in a posterior sensor cluster overlying visual cortex at a corrected *p* = 0.023 (Table 1, line f; Fig. 4*A*, top). For the sensors within this cluster, the mean α-amplitude β across subjects in each condition is plotted in Fig. 4*A* (bottom). Interestingly, white noise input (β = 0) enhanced α-amplitude β compared to the rest (p = 0.0065 [Table 1, line g], paired *t*-test across subjects), and there was a trend effect of stimuli with strong autocorrelation (β = 2) reducing α-amplitude β compared to the rest (*p* = 0.0774 [Table 1, line h]). A control analysis indicated that stimulus β did not significantly influence MEG signal power in the α range. No significant correlation between stimulus β and SCP β was found, nor between stimulus β and ECG α, suggesting that the strength of autocorrelation within SCP and heartbeat dynamics was robust to the range of scale-free stimuli used in this experiment.

### Scale-free neural dynamics predicted behavioral performance

Finally, we investigated whether scale-free neural or physiological dynamics predicted a subject’s behavioral performance in the auditory task. Behavioral performance was assessed by Spearman’s correlation between stimulus β and response β as described above (behavioral ρ). We found that behavioral performance correlated negatively with SCP β (averaged across all conditions) in a group of sensors distributed over frontocentral areas (Fig. 4*B*; *r* = –0.62, *p* < 0.0044 [Table 1, line i]), and positively with α-amplitude β in a group of sensors over left frontotemporal cortices (Fig. 4*C*; *r* = 0.64, *p* = 0.0030 [Table 1, line j]). These results suggest that higher autocorrelation within α-oscillation amplitude fluctuations, and lower autocorrelation within the SCP, predict better discrimination performance on scale-free auditory stimuli. Importantly, neither SCP power nor α-oscillation power correlated with behavioral performance, suggesting that signal power was not a mediating factor between scale-free neural dynamics and behavioral performance. A control analysis further suggested that the above effects are regionally specific: α-amplitude β in the frontocentral area (Fig. 4*B*, left) did not significantly correlate with behavioral performance (*r* = 0.15, *p* = 0.55 [Table 1, line k]), nor did SCP β in the left frontotemporal area (Fig. 4C, left; *r* = –0.32, *p* = 0.19 [Table 1, line l]).

## Discussion

In this study, we investigated the interrelations among scale-free dynamics in the SCP, α-oscillation amplitude fluctuations, and heartbeat dynamics across MEG sensors and subjects and over time within an individual. We further explored their modulation by scale-free dynamic stimuli and tested whether an individual’s scale-free neural dynamics predicted the ability to tell scale-free stimuli apart based on autocorrelation property. Below, we summarize our findings in turn and discuss their implications.

### Interrelations among scale-free neural and physiological dynamics

Across the scalp, a qualitative pattern emerged such that sensors exhibiting stronger autocorrelation (hence, larger power-law exponent β) in the SCP tended to have weaker autocorrelation in the α-oscillation amplitude fluctuations. In addition, SCP β and α-amplitude β were anticorrelated across subjects within a large posterior sensor cluster. This anticorrelation could not be explained by the nested-frequency coupling between SCP and α oscillations, as a control analysis based on simulation suggested that the phase-amplitude coupling between SCP and α oscillations contributes to a positive correlation between their power-law exponents instead. Together, these results suggest that not only do scale-free dynamics exist within both arrhythmic brain activity and amplitude fluctuations of brain oscillations, but these different scale-free neural dynamics are related and follow a systematic antagonistic pattern. Functionally, this anticorrelation may be important for preventing excessive long-range temporal correlation in the brain, such that strong autocorrelation in one type of neural signals impedes the generation of strong autocorrelation in another. Because proper brain functioning requires a balance of sufficient order and flexibility, such anticorrelation may be evidence of a negative feedback mechanism whereby self-organized brain activity is regulated across levels to avoid excessive regularity or overly random fluctuation. The current study does not address the mechanism giving rise to the anticorrelation between SCP β and α-amplitude β across subjects. In particular, it remains unknown whether these two measures have a common mechanism or different mechanisms under common influence, or alternatively, whether one measure influences the other directly or indirectly. Nonetheless, developmental and genetic contributions that have been shown to influence α-amplitude β (Linkenkaer-Hansen et al., 2007; Smit et al., 2011) indicate possible starting points for future investigations to probe.

We further observed that SCP power positively correlated with SCP β across time within an individual, indicating that higher β in the SCP is a result of higher power in the lowest frequencies. This resonates with a previous finding on the pattern of power spectral changes in this frequency range during task performance (He et al, 2010).

Our results reveal a novel relationship between scale-free neural and physiological dynamics, with the strengths of autocorrelation in the SCP and heartbeat dynamics (captured by SCP β and ECG α, respectively) positively comodulating across trials within an individual. Because neither measure was influenced by stimulus condition, this relationship is due to their intrinsic fluctuations over time. By contrast, we did not observe a significant relationship between α-amplitude β and ECG α; such a relationship was reported in Palva et al. (2013) but failed to be reproduced in a later study from the same group (Zhigalov et al., 2015), who reported a correlation between the scaling exponents of δ-oscillation amplitude and heartbeat dynamics; however, they tested many frequency bands and brain systems without correcting for multiple comparisons. In addition, neither of these two previous studies directly recorded ECG, but rather used independent component analysis–extracted component from the MEG recording to substitute for heartbeat signal. Our result with direct ECG recording suggests that fluctuations in slow, arrhythmic neural activity coordinate with heart signals, although the directionality of this influence remains unknown at present. We speculate that a tight correlation between SCP β and ECG α may be because the time scales at which SCP and heartbeat dynamics fluctuate are comparable, both taking place on the order of many seconds (SCP, 0.2–200 s; heartbeat, 4–64 s; compared with α amplitude, 1–10 s). Together, the anticorrelation between SCP β and α-amplitude β and the positive correlation between SCP β and ECG α may suggest the SCP as a central link that connects scale-free neural and physiological dynamics across scales and systems. Nonetheless, neuroanatomical interpretation for the spatial distribution of sensors whose SCP β correlate with ECG α (Fig. 3*E*) would be better informed by future investigations using invasive recordings and/or source reconstruction.

More broadly, it has been shown that the brain exerts strong autonomic influence on and receives feedback from the heart (Craig, 2002; Gray et al., 2007). Previous studies suggest that scale-free heartbeat dynamics may be adaptive, with its long-range temporal correlation serving as a self-organizing mechanism for highly intricate processes that generate fluctuations across wide timescales (Ivanov et al., 1996). Indeed, highly periodic or rigid behaviors may narrow functional responsiveness, as shown by the observation that the breakdown of scale-free heart dynamics and appearance of excessive regularity often accompany pathologies such as severe congestive heart failure (Goldberger et al., 2002).

### Stimulus modulation of scale-free neural dynamics

We observed a systematic modulation of α-oscillation amplitude dynamics by scale-free auditory stimuli, such that α-amplitude β decreased with increasing stimulus β in a posterior sensor cluster overlying occipital cortex. Our stimuli captured a range of stationary and nonstationary patterns, from fractional Gaussian noise to fractional Brownian motion. This result suggests that listening to stimuli that exhibit strong autocorrelation reduces autocorrelation in α amplitude fluctuations in visual regions. A control analysis further suggested that stimulus β had no effect on MEG signal power in the α range. Why should an auditory task affect scale-free neural dynamics in visual regions? Although the underlying mechanisms of this phenomenon require future investigation, a speculative possibility is that higher stimulus β translates into lower α-amplitude β in visual regions owing to cross-modality interaction carried by an inhibitory pathway from auditory cortex to visual cortex (Iurilli et al., 2012).

In contrast, we did not observe a significant correlation between stimulus β and SCP β after cluster-based correction. This negative finding could have several reasons. It is possible that SCP reflects a backbone of brain network structure that remains unperturbed by changes in arousal state (He et al., 2008) or the range of scale-free stimuli used herein. Yet at present, we cannot rule out the possibility that the sample size in the current study was insufficient for detecting an effect in the SCP.

### Prediction of behavioral performance

We found intriguing evidence suggesting that baseline characteristics of scale-free dynamics in the SCP and α-amplitude fluctuations predicted an individual’s performance in discriminating between scale-free auditory stimuli exhibiting different levels of autocorrelation. Better performance correlated with higher α-amplitude β and lower SCP β. Moreover, neither SCP nor α-oscillation power correlated with behavioral performance, suggesting specific behavioral relevance of scale-free parameters. Previous studies have shown that α-amplitude β correlates with long-range temporal correlation in behavioral fluctuations across normal subjects (Palva et al., 2013; Smit et al., 2013). Yet, it is unclear whether longer or shorter autocorrelation in behavioral fluctuations is adaptive. On the other hand, discriminating natural stimuli based on their time-aggregate statistics should confer behavioral advantage in an ecologically natural environment. To our knowledge, this is the first study demonstrating that properties of scale-free neural dynamics predict behavioral performance across normal individuals.

Why should lower SCP β and, conversely, higher α-amplitude β predict better behavioral performance? One possibility is that SCP fluctuations include frequencies an order of magnitude lower than α-amplitude fluctuations (0.005–5 vs. 0.1–1 Hz). Thus, this pattern of result is consistent with the idea that there may be an optimal range of autocorrelation that is most conducive to performing this task: relatively weak autocorrelation in the very long time scales encompassed by the SCP, and relatively high autocorrelation in the comparatively shorter time scales spanned by α-amplitude fluctuations. Higher α-amplitude β might also suggest a state closer to criticality with higher information-processing capacity (Shew et al., 2011; Poil et al., 2012). Tantalizing clues supporting the existence of an optimal range of scale-free neural dynamics exist from studies of clinical populations. For example, breakdown of long-range temporal correlation in θ- and α-oscillation amplitude fluctuations has been observed in depression (Linkenkaer-Hansen et al., 2005), Alzheimer’s disease (Montez et al., 2009), and schizophrenia (Nikulin et al., 2012). On the other hand, abnormally high long-range temporal correlation in β-band amplitude fluctuations is found in seizure-onset areas (Monto et al., 2007).

Finally, in our experiment, the auditory stimuli were constructed such that the scale-free statistic, embodied in the power-law exponent β, is the only difference between categories of auditory tone sequences. All other statistics, including tone duration, pitch range, sequence length, and higher-order statistics (which are random), were identical across stimulus categories (β = [0, 0.2, 0.5, 1.5, 2]). Moreover, behavioral discrimination was carried out on power-law exponent β, not the specific sequence presented; this was ensured by presenting six unique sequences at each β level and asking subjects to make discrimination about β only. Hence, in our task, subjects’ ability to discriminate different auditory tone sequences was specifically related to their ability to process the scale-free statistic of the stimuli, and our findings establish the role of scale-free brain activity in processing scale-free statistics of naturalistic stimuli. On the other hand, our results do not imply that the function of scale-free brain activity is specific to the processing of scale-free or natural stimuli. It is possible that similar correlations may be observed for tasks that do not explicitly require the evaluation of scale-free stimulus statistics. Future studies investigating performance in such tasks will delineate the functional specificity (or generality) of scale-free neural activity.

In summary, we observed novel relationships among scale-free dynamics in distinct components of neural and physiological activity, including the SCP, α-oscillation amplitude fluctuations, and heartbeat dynamics. We further demonstrate that scale-free neural dynamics can be systematically perturbed by scale-free dynamical stimuli that capture second-order statistics (i.e. autocorrelation or power spectrum) of the natural environment. Moreover, the baseline characteristics of scale-free neural dynamics in an individual predict their ability to discriminate scale-free dynamical stimuli based on their autocorrelation property. These results shed light on the complex interrelations among scale-free neural and physiological dynamics at different levels and how they may contribute to adaptive behavior in the natural environment.

## Synthesis

The decision was a result of the Reviewing Editor Lila Davachi and the peer reviewers coming together and discussing their recommendations until a consensus was reached. A fact-based synthesis statement explaining their decision and outlining what is needed to prepare a revision is listed below.

This is a revised version of a manuscript previously reviewed for another journal. The paper reports several novel findings on the relation of scale-free dynamics of slow cortical potentials, alpha oscillations, auditory stimuli and behavior. The revision has improved the manuscript. The following concerns remain:

1) I fully understand the stimuli and that the employed auditory task requires subjects to process the scale-free statistic of the stimuli. However, as explained previously, there is no evidence provided that the reported correlation between performance and neural activity is specific to the scale-free statistic of the stimuli. In other words, the reported correlation between performance and neural activity may also hold for other tasks and stimuli that are completely unrelated to the scale-free statistic of stimuli. I simply miss a control task to establish that the reported effect is specific to processing of naturalistic stimuli. At least, I miss a clear discussion of this lack of a control task to establish specificity.

2) I cannot follow the authors argument that heart-beat analysis based on MEG-ICA is unreliable. From my experience ICA applied to MEG reliably yields robust magnetocardiogram (MCG) signals (typically two ICs). The authors applied ICA to remove heart-artifacts in the present study. Did the authors not find heart-related ICs in all subjects?

3) I suggest to more comprehensively cite previous directly related work by other authors in the introduction. Specifically, I suggest to cite Vanhatalo et al., 2014 and Monto et al., 2007 on the SCP phase alpha amplitude relation and to cite Palva at al., 2013 and Zhigalov et al., 2015 on the relation between scale-free neural activity and heart-rate variability.

4) The reported across-sensor anti-correlation of SCO and alpha beta does not allow for an inference about an intrinsic, i.e. not by chance occurring, relationship between both measures across sensors. This needs to be clearly stated or the analysis needs to be revised. The authors misunderstood my previous comment on this point. The across-sensors result provided by the authors (Fig. 3A and Pearson correlation) does allow for rejecting the Null-hypothesis that SCP beta or alpha beta have inconsistent sensor patterns across subjects. However, this result does not allow for rejecting the critical Null-hypothesis that SCP beta and alpha beta sensor patterns are unrelated, but each consistent, across subjects. Again, consider this latter case: SCP beta and alpha beta sensor patterns are generated by completely unrelated mechanisms and thus have a random correlation (50% below and above 0). If both (mechanistically unrelated) patterns are consistent across subjects (because each pattern has a consistent underlying mechanism), then with sufficient statistical power, i.e. subjects, both pattern will be significantly correlated or anti-correlated half of the time. In other words, if one randomly picks sensor patterns of any two mechanistically unrelated neuronal effects that are consistent across subjects, these distributions will be significantly correlated or anti-correlated half of the time. In order to reject this critical null-hypothesis, one needs to test if the measured magnitude of correlation between patterns could be observed by chance for random patterns with the dimensionality of the measured patterns.

5) As pointed out previously, from the available information it seems as if the authors employed a modulation index based on the index introduced by Tort et al. 2008 (inverted entropy measure) and not based on the index introduced by Canolty et al. 2006 (complex vector length). Thus, I suggest to remove the reference of Canolty et al., 2006. Indeed, in their previous work (He et al., 2010), the authors correctly cite Tort et al. 2008, but not Canolty et al., 2006.
